# Drug-responsive autism phenotypes in the 16p11.2 deletion mouse model: a central role for gene-environment interactions

**DOI:** 10.1038/s41598-020-69130-8

**Published:** 2020-07-23

**Authors:** Emma J. Mitchell, David M. Thomson, Rebecca L. Openshaw, Greg C. Bristow, Neil Dawson, Judith A. Pratt, Brian J. Morris

**Affiliations:** 1 Strathclyde Institute of Pharmacy and Biomedical Sciences, University of Strathclyde, Glasgow, G4 0RE UK; 2 Institute of Neuroscience and Psychology, College of Medical, Veterinary and Life Sciences, University of Glasgow, Sir James Black Building, Glasgow, G12 8QQ UK; 3 Department of Biomedical and Life Sciences, Lancaster University, Lancaster, LA1 4YW UK; 4 Present Address: School of Pharmacy and Medical Sciences, University of Bradford, Bradford, BD7 1DP UK

**Keywords:** Behavioural methods, Neuroscience, Genetics of the nervous system, Social behaviour

## Abstract

There are no current treatments for autism, despite its high prevalence. Deletions of chromosome 16p11.2 dramatically increase risk for autism, suggesting that mice with an equivalent genetic rearrangement may offer a valuable model for the testing of novel classes of therapeutic drug. 16p11.2 deletion (16p11.2 DEL) mice and wild-type controls were assessed using an ethological approach, with 24 h monitoring of activity and social interaction of groups of mice in a home-cage environment. The ability of the excitation/inhibition modulator N-acetyl cysteine (NAC) and the 5-HT_1B/1D/1F_ receptor agonist eletriptan to normalise the behavioural deficits observed was tested. 16p11.2 DEL mice exhibited largely normal behaviours, but, following the stress of an injection, showed hyperlocomotion, reduced sociability, and a strong anxiolytic phenotype. The hyperactivity and reduced sociability, but not the suppressed anxiety, were effectively attenuated by both NAC and eletriptan. The data suggest that 16p11.2 DEL mice show an autism-relevant phenotype that becomes overt after an acute stressor, emphasising the importance of gene-environmental interactions in phenotypic analysis. Further, they add to an emerging view that NAC, or 5-HT_1B/1D/1F_ receptor agonist treatment, may be a promising strategy for further investigation as a future treatment.

## Introduction

Autism is extremely common, affecting males more than females (estimated to affect roughly 4/1,000 boys and 1/1,000 girls)^1,2^, and characterised by communication difficulties, social dysfunction, and repetitive or restricted behaviour patterns, with a high rate of comorbid conditions such as anxiety. There are no available drug treatments for autism. Development of improved treatments will only be enabled by increased understanding of the causes of the disease and how they impact on neurobiology, informed by better preclinical models of facets of the disease.

The genetic architecture of autism is complex^3^. While a large number of common sequence variations increase disease risk, each has only a very small effect individually, and it is the cumulative burden of a range of risk, and protective, gene variants that underlies the aetiological mechanisms ultimately resulting in the manifestation of the common, sporadic disease. However, it is now clear that very rare copy number variants (CNVs), where small numbers of genes are present in one or three, rather than two, copies, substantially increase disease risk. For example, carriers of the deletions of the 16p11.2 locus have dramatically increased risk of autism-spectrum disorders (ASD) and also intellectual disability (8–40x)^4–6^. Interestingly, a high proportion of carriers of the corresponding 16p11.2 *duplication* develop schizophrenia, suggesting that studying the neurobiological impact of CNVs at this locus may be particularly informative. The 16p11.2 deletion is one of the most powerful genetic risk factors for autism^3^.

Various drug classes have been tested in mouse models relevant to ASD, for efficacy in reversing behavioural deficits in social paradigms. These social paradigms typically involve placing unfamiliar pairs of rodents in a novel environment (e.g. open field or 3 chambered apparatus) and recording their interactions over a short timeframe^7^. These relatively simple, high-throughput tests involve separating an animal from its cage mates and placing it in an unfamiliar apparatus. This results in stress-associated social disruption, which in the case of genetically-modified animals may interact with the environmental stressor to reveal a phenotype. Hence the outcomes determined in this testing paradigm may reflect a gene-environmental interaction phenotype, rather than a basal phenotype caused by the model^8^. While this is not necessarily a confound to the disease-relevance of the model, it is important to dissociate the phenotypes that arise directly from the genetic manipulation and those that arise in response to gene-environment interactions, as this offers new insight into the basis of disease symptoms and aetiology, and is important in the context of drug validation and therapeutic relevance.

Abnormal phenotypes in mouse models relevant to ASD have reportedly been improved by statins^9^, Akt/mTOR inhibitors^10^, mGlu5 receptor negative allosteric modulators^11^, mGlu5 receptor positive allosteric modulators^12^^,^^13^, and GABA-B receptor agonists^14–16^. However, these findings have not translated successfully to clinical trials^17–19^; see also^20^. The reasons for this are unclear, but arguably are more likely to relate to the questionable translatability of the behavioural assays employed, rather than the construct validity of the genetic models.

In the specific case of mice engineered to reproduce the 16p11.2 deletion (16p11.2 DEL mice), behavioural deficits have reportedly been normalised by the GABA-B receptor agonist baclofen^21^, a mGlu5 receptor negative modulator^22^, a 5-HT_2A_ receptor antagonist^23^ and a 5-HT_1B/1D/1F_ agonist^24^. The possibility that serotonergic drugs might have therapeutic potential is strengthened by the report of decreased 5-HT turnover in 16p11.2 DEL mice^23^, and that syntenic 16p11.2 deletion restricted to serotonergic neurones in mice is reportedly sufficient to decrease sociability^24^. However, there is also considerable interest currently in the possibility that modifying the balance between glutamatergic excitation and GABAergic inhibition might be a productive strategy. Excitation/inhibition (E/I) balance is thought to be disturbed in autism^25–27^. Accumulating evidence suggests that it is also a feature of mouse models relevant to ASD, including 16p11.2 DEL mice^28^ and neurexin1 knockout mice (Hughes et al., in press). Recent studies have begun to investigate the behavioural effects of agents such as the E/I modulator N-acetyl cysteine (NAC). NAC is primarily an anti-oxidant, but also stimulates the system Xc- cysteine-glutamate antiporter on glial cells, increasing extrasynaptic glutamate^29^ and thereby facilitating stimulation of presynaptic mGlu2 receptors to inhibit glutamate release.

In this study, we assess the behaviour of 16p11.2 DEL mice in an ethological context, using an automated home cage monitoring system which permits group housed animals to be assessed simultaneously for social and locomotor behaviours, and where the impact of acute stress can be separated from baseline behavioural responses^30–33^. In addition, we assess the ability of NAC, as compared to the 5-HT_1B/1D/1F_ agonist eletriptan, to ameliorate the behavioural impact of the 16p11.2 deletion.

## Methods and materials

### Animals

Male mice hemizygous for the 0.44-Mb region of mouse chromosome 7, syntenic to the human 16p11.2 deletion, were generated by Mills and colleagues^30^ (Jackson Laboratory stock No. 013128), and backcrossed onto a C57BL/6 N background to generate experimental mice. It is important to emphasise that, in contrast to some other reports using this genetic modification, the mice in this study have been back-crossed onto the C57BL/6 N background until they are effectively congenic. The 16p11.2 DEL mice, and littermate wild-type (WT) controls, were housed with litter mates in their home cages from weaning. This was to ensure a minimal impact from external environmental factors during the development period from weaning to adulthood which may have influenced their behaviour prior to exposure to the experimental stressor in adulthood. Animals were housed in cages of mixed or same-genotype as previous studies with this line have shown that a range of behaviours including social approach, same-sex social interactions, open field and anxiety-related behaviours were not affected by housing in mixed-genotype versus same-genotype cages^34, 35^. Animals were housed under standard conditions, with food and water *ad libitum*. All work was approved by the University of Glasgow Animal Welfare and Ethics Review Board (AWERB) and conducted in accordance with the UK Animals (Scientific Procedures) Act 1986.

### Home cage monitoring

**Figure 1:**
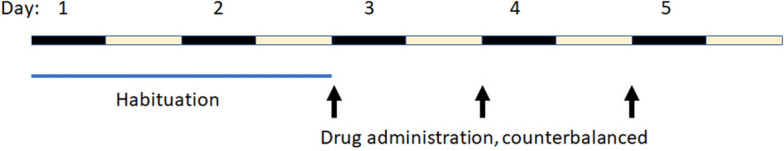
Schedule of home-cage monitoring and drug administration. Days are numbered from introduction to experimental home cage. Dark and light bars represent 12 h dark and light phases**.** Drugs (vehicle, NAC or eletriptan) were administered in a counterbalanced design on days 3–5, at onset of dark period.

14 male mice (7 WT + 7 16p11.2 DEL) were housed in trios or pairs (2 cages of 2 16p11.2 DEL and 1 WT, 1 cage of 3 WT, 1 cage of 3 16p11.2 DEL, and 1 cage of 2 WT) under a reversed light/dark cycle (lights off at 10.00am). Genotypes and numbers in cages were imposed by the genotype/sex configuration of the original litters, to avoid fighting between unfamiliar males put together after weaning. Note that the presence of 2 rather than 3 mice in a cage does not affect locomotor or anxiety measures (e.g. Supplementary Fig. S1). Equally, there was no evidence that the behaviour of mixed genotypes in a cage differed from that of single genotypes (Supplementary Fig. S2). Under isofluorane anaesthesia, a radiofrequency identification (RFID) transponder was implanted subcutaneously into the lower left abdominal quadrant. Groups of 2–3 mice were then placed in Plexiglas IVC cages with Home Cage Analyser (Actual Analytics Ltd, UK)^36,37^ monitoring equipment. Mice were placed in the cages for 1.5 h prior to data acquisition to habituate. Recording then commenced at 10am and proceeded for 72 h. On days 3–5 of testing mice received the following treatments (within subjects counterbalanced design, i.p.): vehicle (50% PEG-400, 10% Solutol HS-15, 40% dH_2_O), NAC (150 mg/kg) and eletriptan (5 mg/kg). All drugs were administered intraperitoneally at an injection volume of 4 ml/kg, 15 min prior to the onset of the dark period (09:45) (Fig. 1). Measures included: total distance travelled (mm), total number of antenna transitions, thigmotaxis, time in centre region of cage, separation (mean Euclidean distance to closest cage-mate (mm)) and social isolation (time spent > 100 mm apart from other cage-mates (s)).

### Data analysis

For the measures of social behaviour (separation and isolation), data from pair-housed mice were excluded, as mice housed in pairs showed greater scores on these measures simply due to there being fewer mice in the cage. Statistical significance was assessed by ANOVA (Minitab), with prior Box-Cox normalisation where data deviated substantially from normality. Mann–Whitney tests were used for specific planned comparisons between groups of particular interest. For the main hypothesised effects, figures also show effect size estimation (estimationstats.com), with mean differences shown as Gardner-Altman estimation plots^38,39^, along with Bayes factors, estimated using JASP^40^, with default (Cauchy) priors. JASP was used to estimate Bayes factors for t-tests (WT mice: vehicle treatment versus 16p11.2 DEL mice: vehicle treatment) or one way repeated-measures ANOVA (16p11.2 DEL mice: vehicle treatment versus NAC treatment versus eletriptan treatment) with post-hoc testing. In all cases, Bayes factors proved to be robust against choice of priors (JASP). Bayes factor magnitudes were interpreted according to standard practice in life sciences research^41^.

### Results

Impaired social functioning is a key feature of autism, but there are limitations in capturing the complexity and multidimensional nature of this domain in rodent models^8^, in part because standard paradigms involve elements of stress responsivity, as behaviour is monitored in a novel environment often between unfamiliar animals, over a limited time frame. We therefore monitored behaviours in group-housed mice in a home cage environment over 5 days^33^. Measures of locomotor activity and anxiety were monitored in parallel.

### Basal locomotor and social activity (habituation, days 1–2)

**Figure 2:**
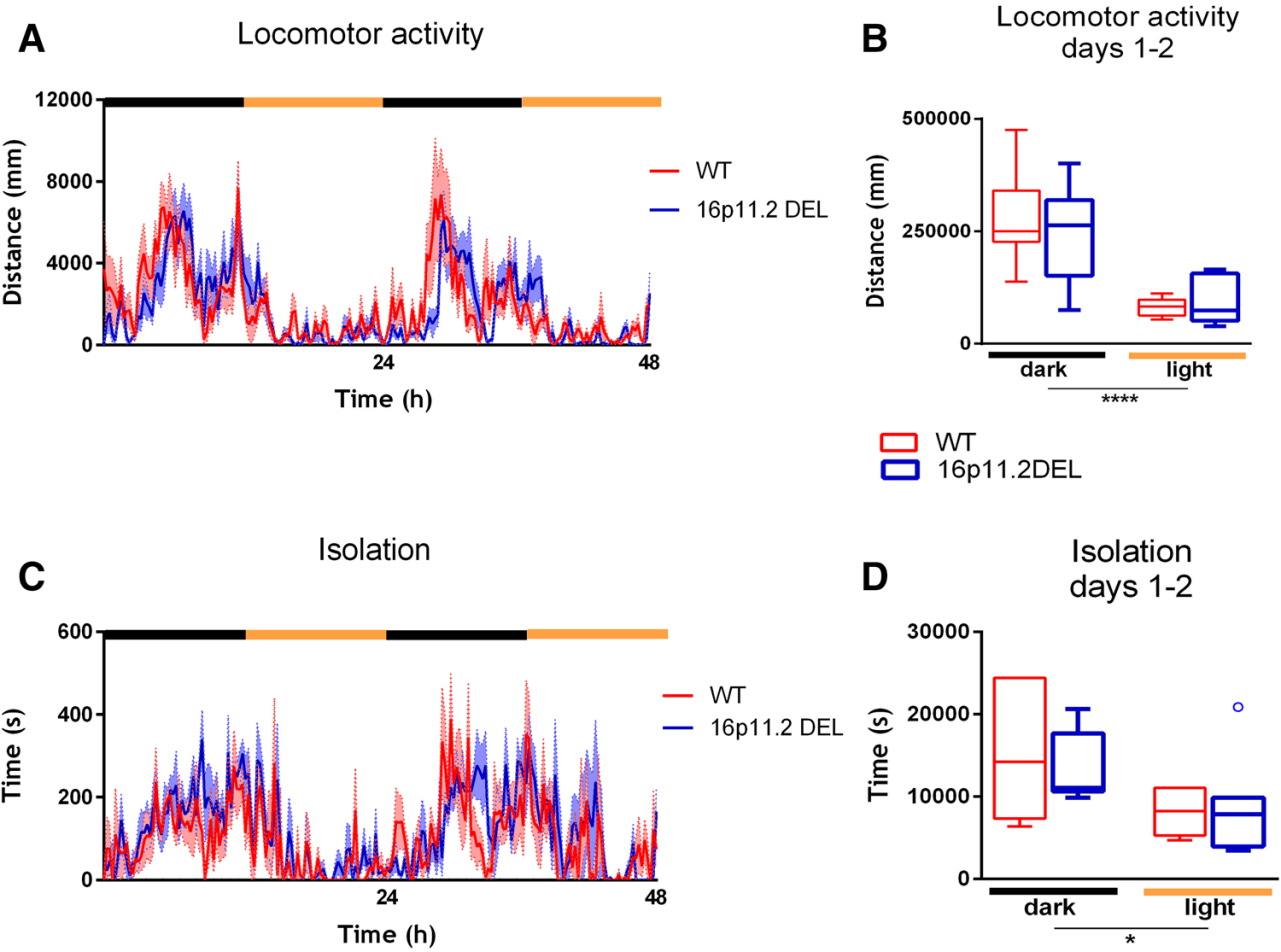
Locomotor activity and isolation from cage-mates during first 2 days. (**A**) Circadian pattern of LMA over days 1–2. Results are shown as mean + /− s.e.m. for 15 min time bins. (**B**) Total LMA shown over days 1 and 2 in dark and light phases. Difference between phases: (F(1, 27) = 34.50; *p* < 0.0001). (**C**) Circadian pattern of isolation over days 1–2. Results are shown as mean + /− s.e.m. for 15 min time bins. (**D**) Total isolation shown over days 1 and 2 in dark and light phases. Difference between phases: (F(1, 27) = 5.30; *p* = 0.03). Box plots show interquartile range with “Tukey” whiskers.

Both WT and 16p11.2 DEL animals exhibited normal circadian activity whereby their active phase was during the dark period (Fig. 2A). Mice were significantly more active during the dark phase versus light phase (F(1, 27) = 34.50; *p* < 0.0001) but there was no genotype effect (*p* = 0.63) (Fig. 2B). This finding is in slight disagreement with previous authors: Horev et al.,^30^ reported mild hyperactivity in DEL animals when placed in novel environment and Arbogast et al.,^31^ reported increased ambulatory activity in DEL mice during the dark phase.

16p11.2 DEL mice showed few signs of social impairment under baseline conditions. Time spent isolated (> 100 mm from all cage-mates) was significantly reduced during the light versus dark phase (F(1, 27) = 5.31; *p* = 0.03) and this was expected, as mice tend to sleep together when the lights are on (Fig. 2C). There was no overall effect of genotype (*p* = 0.78) (Fig. 2D).

There was no evidence that the effects of being moved to the monitoring cage had any interaction with genotype. Both WT and 16p11.2 DEL mice were more active in the first hour after being moved to the monitoring cage, as compared to the equivalent first hour of dark phase on the second habituation day (Supplementary Fig. S3). However, there was no evidence that this, or any of the other behavioural measures, was affected by genotype (Supplementary Fig. S3).

### Drug treatment (days 3–5)

The lack of previously reported hyperlocomotor and reduced sociability phenotypes, under home-cage monitoring conditions, prompted us to examine the response to an acute stressor (handling and injection).

### Locomotor activity

**Figure 3:**
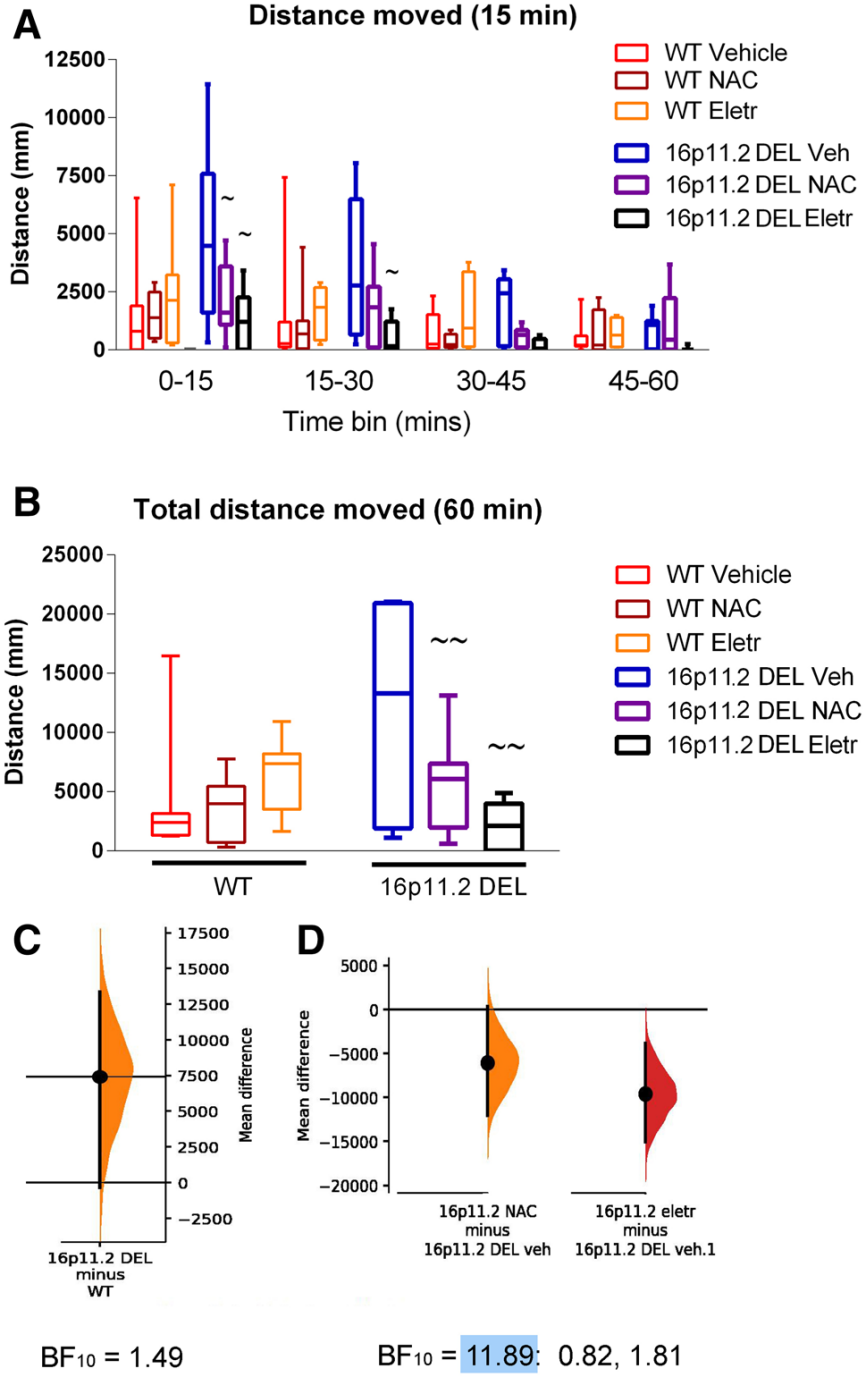
Locomotor activity (distance moved) during first 60 min after injection at start of dark phase on days 3–5. (**A**) Data shown for 15 min time bins. Effect of genotype: (F(1, 167) = 0.02; *p* = 0.88); effect of drug treatment: (F(1, 167) = 6.49, *p* = 0.002; genotype x drug interaction: (F(1, 167) = 12.77, *p* < 0.001; (**B**)–(**D**) Locomotor activity over 60 min after injection on days 3–5. (**B**) Total distance moved: Effect of genotype: (F(1, 167) = 0.02; *p* = 0.88); effect of drug treatment: (F(1, 167) = 6.49, *p* = 0.002; genotype x drug interaction: (F(1, 167) = 12.77, *p* < 0.001. (**C**) The mean difference for total distance moved in 60 min following vehicle injection between WT and 16p11.2 DEL mice, plotted as a bootstrap sampling distribution. The Bayes factor for the alternative hypothesis (BF_10_) of a difference between the vehicle-treated groups, is also shown. (**D**) The mean difference for NAC compared to vehicle, and eletriptan compared to vehicle, groups, in 16p11.2 DEL mice only, are shown in the above Cumming estimation plot. The mean difference is plotted as a bootstrap sampling distribution. The Bayes factors (BF_10_) are also shown for one-way ANOVA of data from 16p11.2 DEL mice (left) (blue shading emphasises strong evidence for an overall effect of drug treatment), and for post-hoc tests of NAC vs vehicle (middle) and eletriptan vs vehicle (right). In all cases, Box plots show interquartile range with “Tukey” whiskers. For effect size plots, the mean difference is depicted as a dot; the 95% confidence interval is indicated by the ends of the vertical error bar. ~*p* < 0.05, ~~*p* < 0.01 vs corresponding vehicle group, same genotype, same time bin (post-hoc Tukey test).

Following injection of vehicle, the distance moved by 16p11.2 DEL mice was substantially greater than that of WT controls for 30–45 min, supporting a stress-induced hyperlocomotor phenotype in these animals (Fig. 3A). By the end of an hour, the increased activity had subsided back to control levels (Fig. 3A). There was no effect of genotype on activity 60–120 min after injection (Supplementary Fig. S4). The elevated activity immediately following injection in 16p11.2 DEL mice was almost completely suppressed when the injection contained either NAC or eletriptan (Fig. 3A–D). Neither NAC nor eletriptan injection modified locomotor activity significantly in WT mice.

When we analysed a different measure of locomotor activity—the number of transitions between different sectors of the home cage, the increased activity in the 16p11.2 DEL mice was less clear (Supplementary Fig. S5), but the effect of the drugs to restore behaviour towards the control condition was still evident (Supplementary Fig. S5).

**Figure 4:**
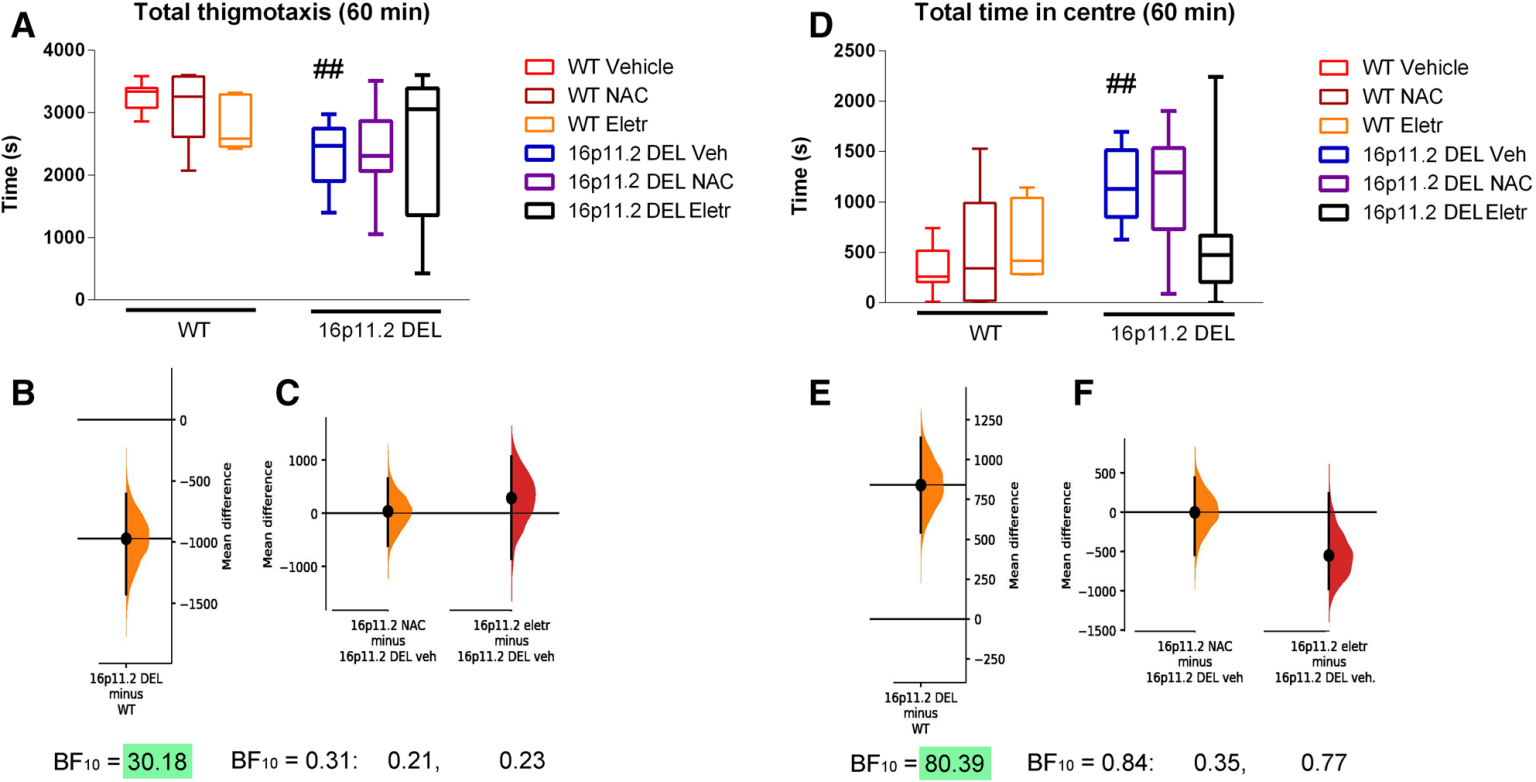
Anxiety measures [thigmotaxis—(**A**)–(**C**)—and time in centre—(**D**)–(**F**)] during first 60 min after injection at start of dark phase. (**A**) Thigmotaxis: Effect of genotype: (F(1, 167) = 3.60; *p* = 0.06); effect of drug treatment: (F(1, 167) = 0.14, *p* = 0.87; genotype x drug interaction: (F(1, 167) = 2.29, *p* = 0.11; ## *p* = 0.003 vs corresponding WT group (one-sided Mann Whitney test). (**B**) The mean difference for thigmotaxis in 60 min following vehicle injection between WT and 16p11.2 DEL mice, plotted as a bootstrap sampling distribution. The Bayes factor for the alternative hypothesis (BF_10_) of a difference between the vehicle-treated groups, is also shown. Green shading emphasises very strong evidence for an effect of genotype. (**C**) The mean difference in thigmotaxis for NAC compared to vehicle, and eletriptan compared to vehicle, groups, in 16p11.2 DEL mice only, are shown in the above Cumming estimation plot. The mean difference is plotted as a bootstrap sampling distribution. The Bayes factors (BF_10_) are also shown for one-way ANOVA of data from 16p11.2 DEL mice (left), and for post-hoc tests of NAC vs vehicle (middle) and eletriptan vs vehicle (right). (**D**) Total time in centre zone**:** Effect of genotype: (F(1, 167) = 3.65; *p* = 0.058); effect of drug treatment: (F(1, 167) = 1.26, *p* = 0.29; genotype x drug interaction: (F(1, 167) = 3.38, *p* = 0.037; ## *p* = 0.003 vs corresponding vehicle group, same genotype (one-sided Mann Whitney test). (**E**) The mean difference for time in centre in 60 min following vehicle injection between WT and 16p11.2 DEL mice, plotted on the right as a bootstrap sampling distribution. The Bayes factor for the alternative hypothesis (BF_10_), of a difference between the vehicle-treated groups, is also shown. Green shading emphasises very strong evidence for an effect of genotype. (**F**) The mean difference for time in centre, for NAC compared to vehicle, and eletriptan compared to vehicle, groups, in 16p11.2 DEL mice only, are shown in the above Cumming estimation plot. The mean difference is plotted as a bootstrap sampling distribution. The Bayes factors (BF_10_) are also shown for one-way ANOVA of data from 16p11.2 DEL mice (left),and for post-hoc tests of NAC vs vehicle (middle) and eletriptan vs vehicle (right). In all cases, Box plots show interquartile range with “Tukey” whiskers. For effect size plots, the mean difference is depicted as a dot; the 95% confidence interval is indicated by the ends of the vertical error bar.

We also analysed behaviours thought to be related to anxiety—thigmotaxis, and time spent in the centre region of the home cage. Following the injection of vehicle, 16p11.2 DEL mice showed a very clear anxiolytic response, as demonstrated by significantly decreased thigmotaxis, and increased total time in the centre of the cage (Fig. 4A,B,D,E). The inhibitory effect of the drugs was less clear here, with no strong evidence that they were able to normalise the responses (Fig. 4A,C,D,F). In addition, both drugs did not modify anxiety-like behaviour in WT mice.

**Figure 5:**
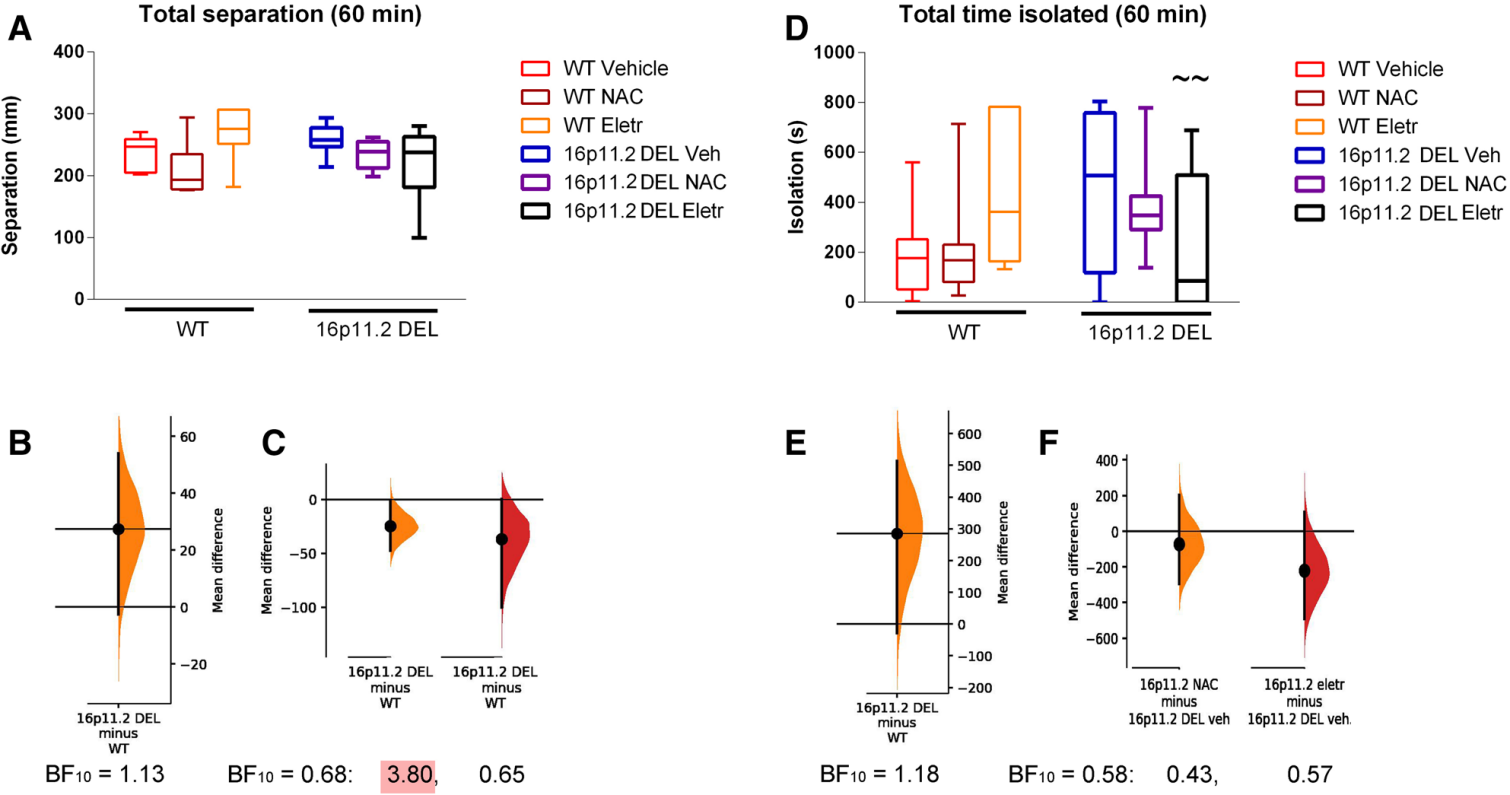
Social interaction measures [distance of separation—(**A**)–(**C**)—and time spent isolated—(**D**)–(**F**)] during first 60 min after injection at start of dark phase. (**A**) Total separation: Effect of genotype: (F(1, 143) = 0.18; *p* = 0.67); effect of drug treatment: (F(1, 143) = 0.91, *p* = 0.41; genotype x drug interaction: (F(1, 143) = 3.14, *p* = 0.047. (**B**) The mean difference for separation in 60 min following vehicle injection between WT and 16p11.2 DEL mice, plotted as a bootstrap sampling distribution. The Bayes factor for the alternative hypothesis (BF_10_) of a difference between the vehicle-treated groups, is also shown. (**C**) The mean difference for separation for NAC compared to vehicle, and eletriptan compared to vehicle, groups, in 16p11.2 DEL mice only, are shown in the above Cumming estimation plot. The mean difference is plotted as a bootstrap sampling distribution. The Bayes factors (BF_10_) are also shown for one-way ANOVA of data from 16p11.2 DEL mice (left), and for post-hoc tests of NAC vs vehicle (middle) and eletriptan vs vehicle (right); red shading emphasises moderate evidence for an effect of NAC. (**D**) Time isolated**:** Effect of genotype: (F(1, 143) = 0.61; *p* = 0.44); effect of drug treatment: (F(1, 143) = 0.70, *p* = 0.50; genotype x drug interaction: (F(1, 143) = 6.96, *p* = 0.001; ~~*p* < 0.01 vs corresponding vehicle group, same genotype (post-hoc Tukey test). (**E**) The mean difference for time spent isolated in 60 min following vehicle injection between WT and 16p11.2 DEL mice, plotted on the right as a bootstrap sampling distribution. The Bayes factor for the alternative hypothesis (BF_10_), of a difference between the vehicle-treated groups, is also shown. (**F**) The mean difference for time isolated NAC compared to vehicle, and eletriptan compared to vehicle, groups, in 16p11.2 DEL mice only, are shown in the above Cumming estimation plot. The mean difference is plotted as a bootstrap sampling distribution. The Bayes factors (BF_10_) are also shown for one-way ANOVA of data from 16p11.2 DEL mice (left), and for post-hoc tests of NAC vs vehicle (middle) and eletriptan vs vehicle (right). In all cases, Box plots show interquartile range with “Tukey” whiskers. For effect size plots, the mean difference is depicted as a dot; the 95% confidence interval is indicated by the ends of the vertical error bar.

When we analysed measures of sociability—the mean separation between mice in the home cage, and the time spent isolated from other mice in the home cage, there was a trend towards reduced sociability in the 16p11.2 mice. The effect of the drugs to restore behaviour towards the control condition was still evident (Fig. 5). Interestingly, eletriptan showed a trend towards increasing isolation in WT mice, that was significant in the 2^nd^ hour after injection (Supplementary Fig. S4).

## Discussion

A number of previous reports have documented hyperactivity, increased or reduced anxiety, and unchanged or reduced sociability, in 16p11.2 DEL mice^21,30,31,42,43^. In all cases, assessments have been made under conditions of some stress to the mice—with the tests involving experimenter handling, exposure to a novel environment, or injection, or social separation or exposure to unfamiliar conspecifics, depending on the task. Hence the resulting phenotype of the previous data potentially represents a combination of genetic and environmental factors. Here we delineate these factors by reporting the basal phenotypes in a relatively stress-free home-cage environment using a continuous monitoring system, and also the effect of an acute injection stressor, to parse these effects in the behavioural phenotypes seen in 16p11.2 DEL mice. In addition, we have characterised the ability of two drugs of interest with respect to future therapeutic options for autism—NAC and eletriptan—to normalise the stress-induced phenotypes observed in 16p11.2 DEL mice, showing dissociable effects on hyperactivity, anxiety-like behaviour and sociability. Both NAC and eletriptan are rapidly absorbed in rodents and other species^44–46^, allowing the assessment of their effects on the post-injection phenotypes observed. Both drugs are also completely cleared by 24 h after administration^44,46,47^, so no carry-over effects are expected in our counter-balanced repeated measures design.

The within subjects, counterbalanced, repeated measures design is a strength of our study, allowing reductions in sample size while maintaining statistical power. For example, a retrospective power analysis from the social isolation data obtained, using Glimmpse^48^, estimated a power of 0.83 to detect a genotype x drug interaction at *p* < 0.05 by ANOVA. Further, in accordance with recent and evolving recommendations^38,39,49^, we include information on the confidence intervals for the main effects, and also some preliminary Bayesian analysis. The use of Cauchy priors for the Bayesian analysis might be seen as over-conservative, considering the previous reports of hyperactivity and hyposociability phenotypes in 16p11.2 DEL mice^21,30,31,50^. While our results clearly align with these previous reports, due to the novel use of ethological behaviours in this study, we preferred to regard the results as entirely distinct from previous work, although in fact the Bayes factors obtained turned out to be relatively robust against varying the prior odds. For example, for the social measures in vehicle-injected mice (separation distance and isolation time), an informed prior based on existing literature with this genetic mutation^30,31,43^ (mean 1.0, std 0.307) yields BF10 values of 3.6 and 3.8 respectively (moderate evidence for a genotype effect).

We have examined the effect of an acute stressor in this study. It will be of great interest in future work to compare the effects of some of the widely-used chronic stress models on the phenotype of this and other mouse genetic models of aspects of ASD.

### Comparison with previous research in 16p11.2 DEL mice

The original report of this strain demonstrated a transient increase in locomotor activity relative to controls, lasting for less than 2 h after transfer to a novel environment^30^. This seems broadly equivalent to our current observations. From testing in a novel environment and/or after an injection, others have observed rather subtle hyperlocomotion^21,31,42,43^, no obvious locomotor phenotype^23,50^, or decreased locomotor activity^51^ in 16p11.2 DEL mice. In another study, Portman et al.^35^ reported increased home cage activity and decreased activity in a novel environment. The reasons for this variability are not clear, although many of these studies used mice maintained on a mixed background, which inevitably leads to increased variability in complex behaviours. It may also be an indication that this phenotype is rather subtle. Our data suggest a primary role in the interaction between environmental stress and the 16p11.2 DEL genotype that may also contribute to the variability of these phenotypes under different environments. Interestingly, the corresponding mice with a duplication of the same region show a *hypo*locomotor phenotype, both in home cage and in a novel environment^30,31,52^. Hence if there is a gene dosage effect in this phenotype, the 16p11.2 DEL mice would be predicted to show elevated activity. We indeed detect increased locomotor activity after an acute stressor, although the effect is transient (Fig. 3). This emphasises the extent to which particular details of behavioral testing procedures (*i.e.* degree of environmental stressor) can influence the results obtained. Furthermore, the data highlight the fundamental need for the application of emerging behavioural testing strategies, such as those used in this study, that allow for the parsing of genetic and environmental factors in the context of determining translationally-relevant phenotypes in genetic mouse models relevant to neurodevelopmental disorders.

While we detect genotype effects on locomotion and anxiety measures, the less clear effects of genotype on sociability measures suggest that reduced sociability is a less overt phenotype associated with 16p11.2 deletion. Indeed, most of the previous studies failed to detect altered social behaviours in 16p11.2 DEL mice (using a novel environment, 3 chamber test)^30,31,35,43^. Other studies have detected social dysfunction, albeit using mice on a mixed background^21,50^. Our data are indicative of subtle social dysfunction that is revealed under conditions of acute stress. Importantly, assessing social interaction between littermates in the home cage environment allows us to characterise sociability in the ethological context of established social hierarchies that exist between mice rather than the sociable behaviour displayed in the context of a novel social interaction with an unfamiliar conspecific (as employed in the 3 chamber test). As mice usually display higher levels aggression in the context of novel social interactions, with lower levels in the context of established hierarchies in the home cage, the potentially confound of this stressor is removed by home cage monitoring of littermate sociability. Future studies will allow the application of both the home cage and 3 chamber social testing paradigms to assess the role of stress and social familiarity in the sociability deficits seen in 16p11.2 DEL, and in other mouse models relevant to autism. It will be of interest to investigate whether exposure to alternative /more sustained stressors produces a larger impact on the social deficits observed.

In terms of the anxiolytic phenotype, other studies of 16p11.2 DEL mice have reported decreased anxiety^21,31,43^, no change^42,50^ or increased anxiety^51^. Again this lack of consistency may at least partially reflect the use of mice on a mixed background in many cases, or the interaction between different stressors in the induction of this phenotype, as supported by our own findings.

### Contribution of individual genes within the 16p11.2 region

There is a paucity of information concerning the influence of specific genes within the 16p11.2 CNV on behaviour. *Kctd13* gene knockout in mice reportedly does not affect locomotion, anxiety or social behaviour^53^. However, mice with a genetic deletion specifically of the *Taok2* gene exhibit hyperactivity and reduced anxiety in a novel environment^54^. Hence it seems likely that *Taok2* gene haploinsufficiency contributes to these phenotypes observed in the current study, especially since reduced JNK signalling (the downstream target of TAOK2) also decreases anxiety and increases locomotion^55,56^. Increased locomotor activity is seen in *Mapk3* knockout mice^57,58^, so decreased ERK1 activity due to hemi-deletion of the *Mapk3* gene in 16p11.2 DEL mice may also contribute to the hyperlocomotor phenotype.

### Restorative effects of NAC

Perturbed E/I balance is thought to be a core neurobiological feature of autism^25–27^, detectable also in various genetic mouse models of autism^28^. Indeed, optogenetic correction of E/I balance restores normal locomotor and social behaviour in *CNTNAP2* knockout mice, another mouse genetic model of aspects of autism^59^. In vitro studies suggest increased E/I ratios in hippocampus from 16p11.2 DEL mice^60^, as do in vivo studies in cerebral cortex^28^. Our data illustrate an ability of acute NAC administration, at a dose previously shown to reduce CNS glutamate levels^61^, to attenuate the increased locomotor response to injection seen in the 16p11.2 DEL mice. This is quite a striking result, and may reflect a strong link between E/I balance and locomotor activity, since system Xc–deficient mice (system Xc- being the target of NAC) show abnormal locomotion^62^. While a trend for NAC to attenuate the reduced sociability of 16p11.2 mice was observed, no effect of NAC was detected on the reduced anxiety-like behaviour manifest by these mice. A previous study reported that the same dose of NAC in mice decreases locomotion, and increases anxiety-like behaviour (decreased centre time in open field)^61^, while Xc–deficient mice show reduced anxiety, as reflected in increased centre time in the open field arena^62^. The lack of effect of NAC on anxiety measures in 16p11.2 DEL mice is slightly surprising in that context. It may be that the mechanisms underlying the reduced anxiety, which is not considered a core symptom of ASD, are distinct from those underlying the reduced sociability and the increased locomotor activity. Alternatively, the beneficial effects of NAC may not be due to lowering of glutamatergic activity. Abnormal ERK and CREB activity is a feature of many autism-related mouse models, and also of some patients^63–65^, and NAC can modulate this pathway effectively^66^, in addition to its well-known anti-oxidant properties.

Whatever the mechanisms involved, our data support further investigation of NAC in preclinical models of autism. Small scale clinical studies have noted some improvements in individuals with ASD with NAC, including potential effects on stereotypies and social cognition^67,68^.

### Restorative effects of eletriptan

Panzini et al. reported evidence for decreased 5-HT turnover in the CNS of 16p11.2 DEL mice^23^, suggesting that serotonergic hypofunction may contribute to the phenotypes seen in these animals. Therefore we tested the ability of the 5-HT_1B/D/F_ agonist to restore these deficits. We found that eletriptan treatment effectively reversed the stress-induced hyperactivity seen in 16p11.2 DEL mice with no effect on the stress-induced reduced anxiety-like and sociability deficits seen in these animals.

5-HT1B agonists increase mouse locomotor activity (although locomotion is essentially normal in 5-HT_1B_ receptor knockout mice)^69^. We saw a trend for eletriptan to increase locomotion in WT mice (Fig. 3A). Our evidence that eletriptan could suppress the hyperlocomotion observed after injection in the 16p11.2 DEL mice was clear. In view of the tendency for 5-HT_1B_ agonists to enhance locomotion in WT mice, this is more likely to reflect an action of the drug on 5-HT_1D_ or 5-HT_1F_ receptors, neither of which are well-studied in terms of their behavioural effects in rodents.

5-HT_1B_ knockout mice do however show reduced thigmotaxis in an open field^69^. Consistent with this, 5-HT_1B/1D_ agonists are generally considered to be anxiogenic, so the greatest efficacy of eletriptan was expected to be seen in the anxiolytic phenotype. However, we saw little evidence for an anxiogenic effect of eletriptan in WT mice (Fig. 4A,B), or of an ability to normalise the anxiolytic phenotype observed after an injection in the 16p11.2 DEL mice, indicating a more complex role for eletriptan in anxiogenesis.

It was interesting to note that eletriptan increased social isolation in WT mice in the 2nd hour after injection (Supplementary Fig. S4)—a time when eletriptan will still be present in the CNS^44^. This is to some extent expected, as 5-HT_1B/1D_ agonists increase social fear in rodents and humans^70,71^, but the effect is not observed in 16p11.2 DEL mice. Syntenic 16p11.2 deletion restricted to serotonergic neurones in mice reportedly decreases sociability—an effect that can be rescued by stimulation of 5-HT_1B_ receptors^24^. The present finding of an ability of electriptan to alleviate a social behavioural deficit in 16p11.2 DEL mice suggests further investigation of 5-HT subtype specific agonists in ASD is warranted.

## Conclusions

Clinically, the appearance and exacerbation of autistic symptoms are associated with acute or chronic stress^72–74^. An acute stress revealed locomotor, anxiolytic and impaired social behaviours in 16p11.2 DEL mice, that were not present under low stress conditions, suggesting that environmental factors such as stress interact with the genetic variant to reveal a disorder-relevant phenotype. Autistic subjects however tend to be hyperanxious rather than the opposite^75^, so the translational significance of the anxiolytic response seen in 16p11.2 DEL mice is unclear. NAC and eletriptan showed intriguing efficacy to attenuate the more disease-relevant responses. Our data support evolving concepts that the correction of an unbalanced relationship between excitation and inhibition in the CNS, or, most strikingly, stimulation of 5-HT_1B/1D/1F_ receptors to counteract serotonergic hypofunction, could be important future therapeutic strategies. In particular these data are important in a translational context given that drug-effectiveness was revealed in conditions where a stressor impacted on the behavioural outcome.

## Supplementary information

Supplementary Information.
