# Supplementary material for: Drug-responsive autism phenotypes in the 16p11.2 deletion mouse model: a central role for gene-environment interactions
Source: Sci Rep. 2020 Jul 23;10:12303. doi: 10.1038/s41598-020-69130-8 (PMC7378168; doi:10.1038/s41598-020-69130-8)
Supplement: Supplementary file 1 — Supplementary Information. [file 41598_2020_69130_MOESM1_ESM.pdf]

Drug-responsive autism phenotypes in the 16p11.2 deletion mouse model: a central role for gene-environment interactions

Emma J. Mitchell, David M. Thomson, Rebecca L.Openshaw, Greg C.Bristow, Neil Dawson, Judith A. Pratt, Brian J. Morris

Supplementary Information

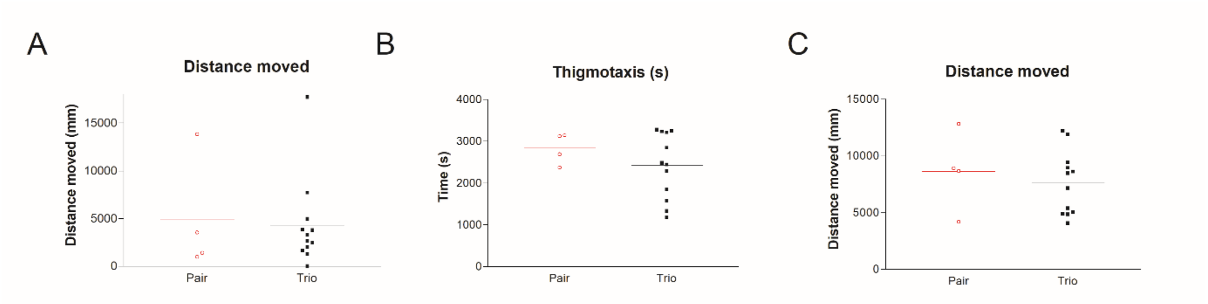

Supplementary Figure S1

Lack of significant impact of pair vs trio housing on locomotor and anxiety measures. A, C) Locomotor activity, and **B)** thigmotaxis, in mice housed as pairs or trios. Example data during the dark phase. A) 2nd hour, day1 p=0.83 (t-test); B) 2nd hour, day1 p=0.33 (t-test); 1st hour, day2 p=0.55 (t-test).

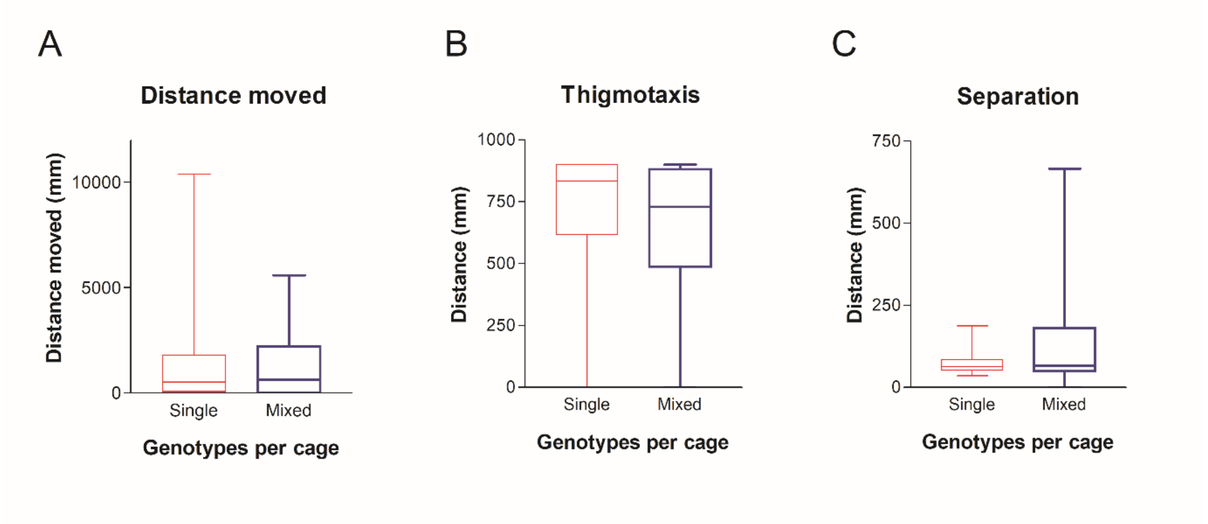

Supplementary Figure S2

Lack of significant impact of single genotype vs mixed genotype housing on locomotor, anxiety and social measures. A) Locomotor activity, **B)** thigmotaxis, and **C)** separation, in mice housed in groups of a single genotype or mixed genotype (WT and 16p11.2 DEL). Example data during the first hour of the dark and light phase on the habituation days, shown as the overall average values per 15 minute time bin.

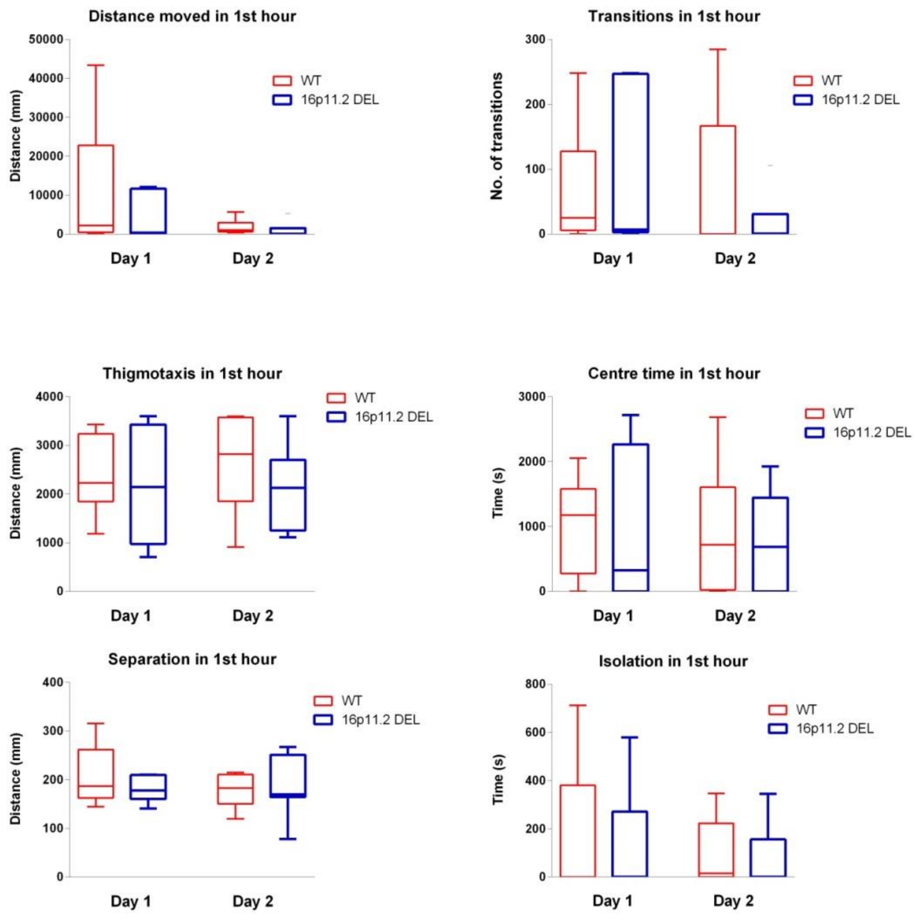

Supplementary Figure S3

Locomotor measures (top panels), anxiety measures (middle panels), and social measures (bottom panels) in WT and 16p11.2 DEL mice in the first hour of the dark phase, on initial transfer to the monitoring cage on day 1, and in the equivalent period 24h later after 1 day of habituation. No significant effects of genotype are observed.

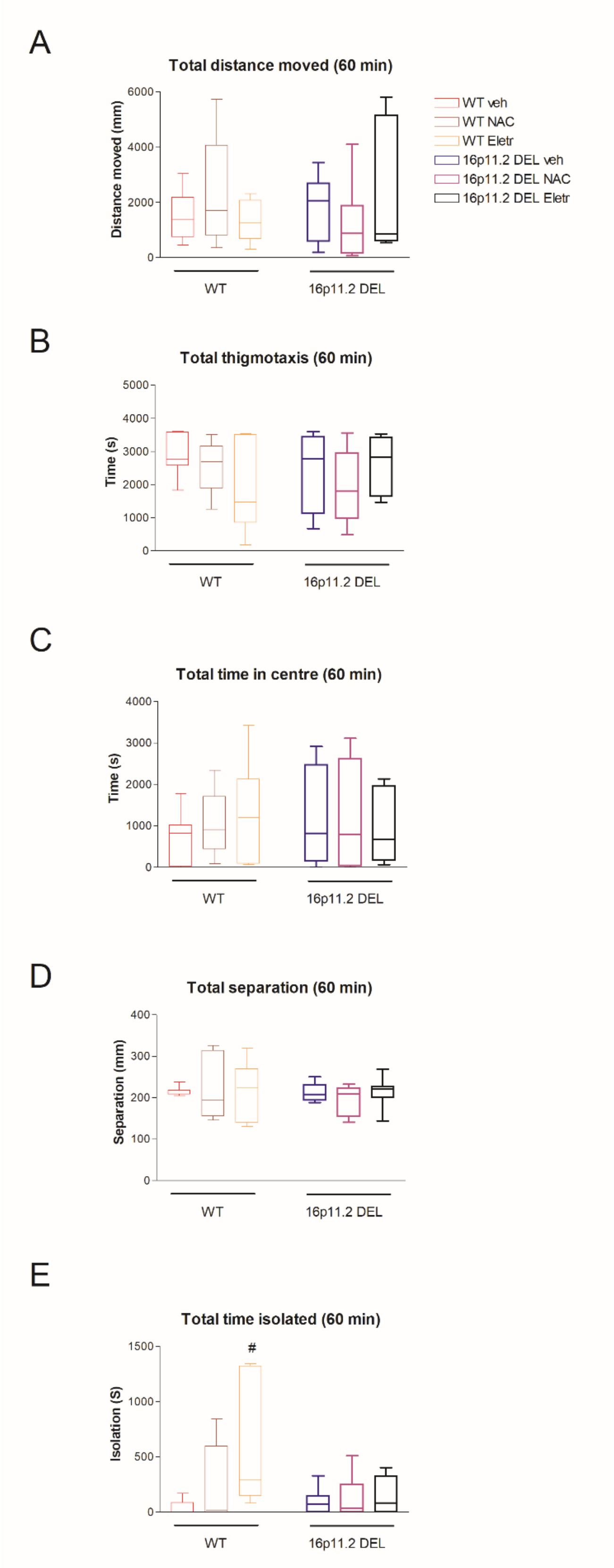

Supplementary Figure S4

No effect of genotype on activity in the 2nd hour (60-120 min) after injection. Locomotor activity **A**), anxiety (thigmotaxis, **B**, and time in centre, **C**) and social phenotypes (separation **D**, and isolation, **E**) were assessed after injection of vehicle, NAC or eletriptan. There were no significant effects of genotype or drug, or any genotype x drug interactions, by ANOVA. # $p<0.05$ Mann-Whitney test vs WT vehicle group.

Supplementary Figure S5

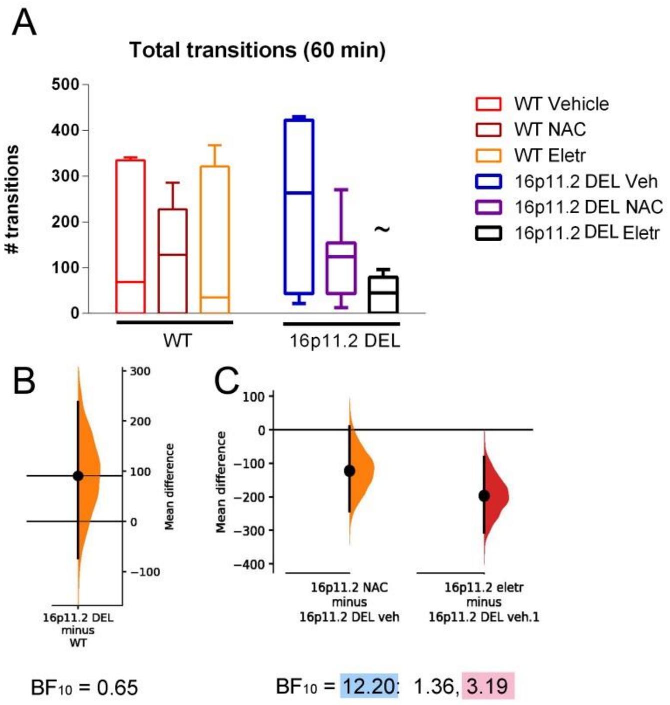

Locomotor activity measure (Transitions between sectors) during first 60 minutes after injection at start of dark phase. A) Number of transitions**:** Effect of genotype: (F (1, 167) = 0.01; p=0.91); effect of drug treatment: (F (1, 167) = 3.65, p=0.029; genotype x drug interaction: (F (1, 167) = 4.41, p=0.014; ~ p<0.07 vs corresponding vehicle group, same genotype (post-hoc Tukey test). **B)** The mean difference for number of transitions in 60 minutes following vehicle injection between WT and 16p11.2 DEL mice, plotted on the right as a bootstrap sampling distribution. The Bayes factor for the alternative hypothesis (BF_{10}), of a difference between the vehicle-treated groups, is also shown. **C)** The mean difference for NAC compared to vehicle, and eletriptan compared to vehicle, groups, in 16p11.2 DEL mice only, are shown in the above Cumming estimation plot. The mean difference is plotted as a bootstrap sampling distribution. The Bayes factors (BF_{10}) are also shown for one-way ANOVA of data from 16p11.2 DEL mice (left) (blue shading emphasises strong evidence, red shading indicates moderate evidence, for an overall effect of drug treatment), and for post-hoc tests of NAC vs vehicle (middle) and eletriptan vs vehicle (right); red shading emphasises moderate evidence for an effect of eletriptan. In all cases, Box plots show interquartile range with "Tukey" whiskers. For effect size plots, the mean difference is depicted as a dot; the 95% confidence interval is indicated by the ends of the vertical error bar.
